# Impact of a web-based tool (WebCONSORT) to improve the reporting of randomised trials: results of a randomised controlled trial

**DOI:** 10.1186/s12916-016-0736-x

**Published:** 2016-11-28

**Authors:** Sally Hopewell, Isabelle Boutron, Douglas G. Altman, Ginny Barbour, David Moher, Victor Montori, David Schriger, Jonathan Cook, Stephen Gerry, Omar Omar, Peter Dutton, Corran Roberts, Eleni Frangou, Lei Clifton, Virginia Chiocchia, Ines Rombach, Karolina Wartolowska, Philippe Ravaud

**Affiliations:** 1 Oxford Clinical Trials Research Unit, Nuffield Department of Orthopaedics, Rheumatology and Musculoskeletal Sciences, University of Oxford, Oxford, UK; 2 Centre for Statistics in Medicine, Nuffield Department of Orthopaedics, Rheumatology and Musculoskeletal Sciences, University of Oxford, Oxford, UK; 3 Centre d’Epidémiologie Clinique, Paris Descartes University, Paris, France; 4 INSERM UMR 1153 equipe Methods, Paris Descartes University, Paris, France; 5 Queensland University of Technology (QUT), Queensland, Australia; 6 Ottawa Hospital Research Institute, Ottawa, Canada; 7 Mayo Clinic, Minnesota, USA; 8 University of California, Los Angeles, USA

**Keywords:** Randomised controlled trial, CONSORT, Transparency, Reporting

## Abstract

**Background:**

The CONSORT Statement is an evidence-informed guideline for reporting randomised controlled trials. A number of extensions have been developed that specify additional information to report for more complex trials. The aim of this study was to evaluate the impact of using a simple web-based tool (WebCONSORT, which incorporates a number of different CONSORT extensions) on the completeness of reporting of randomised trials published in biomedical publications.

**Methods:**

We conducted a parallel group randomised trial. Journals which endorsed the CONSORT Statement (i.e. referred to it in the Instruction to Authors) but do not actively implement it (i.e. require authors to submit a completed CONSORT checklist) were invited to participate. Authors of randomised trials were requested by the editor to use the web-based tool at the manuscript revision stage. Authors registering to use the tool were randomised (centralised computer generated) to WebCONSORT or control. In the WebCONSORT group, they had access to a tool allowing them to combine the different CONSORT extensions relevant to their trial and generate a customised checklist and flow diagram that they must submit to the editor. In the control group, authors had only access to a CONSORT flow diagram generator. Authors, journal editors, and outcome assessors were blinded to the allocation. The primary outcome was the proportion of CONSORT items (main and extensions) reported in each article post revision.

**Results:**

A total of 46 journals actively recruited authors into the trial (25 March 2013 to 22 September 2015); 324 author manuscripts were randomised (WebCONSORT *n* = 166; control *n* = 158), of which 197 were reports of randomised trials (*n* = 94; *n* = 103). Over a third (39%; *n* = 127) of registered manuscripts were excluded from the analysis, mainly because the reported study was not a randomised trial. Of those included in the analysis, the most common CONSORT extensions selected were non-pharmacologic (*n* = 43; *n* = 50), pragmatic (*n* = 20; *n* = 16) and cluster (*n* = 10; *n* = 9). In a quarter of manuscripts, authors either wrongly selected an extension or failed to select the right extension when registering their manuscript on the WebCONSORT study site. Overall, there was no important difference in the overall mean score between WebCONSORT (mean score 0.51) and control (0.47) in the proportion of CONSORT and CONSORT extension items reported pertaining to a given study (mean difference, 0.04; 95% CI −0.02 to 0.10).

**Conclusions:**

This study failed to show a beneficial effect of a customised web-based CONSORT checklist to help authors prepare more complete trial reports. However, the exclusion of a large number of inappropriately registered manuscripts meant we had less precision than anticipated to detect a difference. Better education is needed, earlier in the publication process, for both authors and journal editorial staff on when and how to implement CONSORT and, in particular, CONSORT-related extensions.

**Trial registration:**

ClinicalTrials.gov: NCT01891448 [registered 24 May 2013].

## Background

Published articles reporting on the methodology and results of clinical trials are most often, for all readers, the only way to know how a study was conducted and what the results were. These articles must present accurate, unbiased, and transparent information concerning the methodology and conduct of the trial for the reader to assess the validity, generalizability, and applicability of the trial results.

Many studies have evaluated the quality of reporting in randomised trials in almost every clinical specialty and subspecialty [1–8]. In nearly every study, the results indicate that many crucial methodological elements are not reported in published reports of randomised trials. For example, in a sample of 616 randomised trials indexed in PubMed in December 2006, the primary endpoint was not defined in 47% of trials, the method used to generate the sequence of randomisation was not reported in 66%, the method used to conceal allocation was not reported in 75%, and the sample size calculation was not reported in 56% [8]. When the tested interventions or studied populations are insufficiently described, reproducing these interventions is impossible [9], as is assessing the population to which the results may apply. Lack of transparency is a major limiting factor for clinicians wanting to translate best evidence into best practice. It is also a major problem for scientists who perform systematic reviews and meta-analyses as some published trials may have to be excluded because of missing information [10, 11].

Lack of transparency [12–14] is mainly the responsibility of the authors of articles, but peer reviewers and journal editors should ensure that the results are based on an appropriate methodology. The CONSORT (CONsolidated Standards of Reporting Trials) Statement, published in 1996 and updated in 2001 and 2010 [15, 16], was designed to improve the transparency and quality of the reporting in clinical trials. It comprises a checklist of 25 items and a flow chart that allows visualisation of the flow of patients through the study, from recruitment to the analysis of the results. This recommendation, endorsed by a considerable number of medical journals, informs not only authors, but also reviewers and editors, about which information should be included in articles to facilitate critical judgment and interpretation of results. A recent systematic review showed that endorsement of the CONSORT Statement by a journal was associated with an improvement in the quality of reporting of randomised trials [17].

Although the CONSORT Statement applies to all randomised trials, it is primarily appropriate for superiority trials with two parallel treatment arms and individual randomisation. Several extensions of the CONSORT Statement have been developed to specify the additional information needed in reports of trials with different designs (e.g. non-inferiority [18], cluster randomised [19], and pragmatic trials [20]) or for specific interventions (e.g. non-pharmacological treatments [21], acupuncture, [22] and herbal therapy [23]). Each of these extensions includes a list of items modified from the original CONSORT Statement or new items that need to be addressed when reporting these trials. However, proliferation of these extensions makes their application difficult for a specific trial as it involves combining items from the main CONSORT checklist with those from one or more extensions. This could be cumbersome and difficult to apply in practice and so CONSORT may have limited impact on the reporting quality of these trials.

The objective of this study was to evaluate the impact of a simple web-based tool (called WebCONSORT, which incorporates the main CONSORT checklist and different CONSORT extensions) on the completeness of reporting of randomised trials published in biomedical journals. WebCONSORT allows authors to obtain a customised CONSORT checklist and flow diagram specific to their trial design and type of intervention. Our hypothesis was that this tool would allow optimal use of the CONSORT Statement and its extensions, thus leading to an improvement in the transparency of articles related to randomised trials.

## Methods

### Design

We conducted a multi-journal, two-arm parallel group, randomised trial to assess the impact of the WebCONSORT tool compared to a control intervention on the completeness of reporting of randomised trials submitted to biomedical journals. The study obtained ethics approval from the University of Oxford Central Research Ethics Committee, Oxford, UK (MSD-IDREC-C1-2012-89) and is registered on ClinicalTrials.gov (NCT01891448).

### Journal participants

To be eligible for inclusion, journals were required to (1) endorse the CONSORT Statement (assessed via journal Instruction to Authors and as listed on the CONSORT website: www.consort-statement.org); (2) not actively implement the CONSORT Statement (defined as requiring authors to submit a completed CONSORT checklist alongside their manuscript at the time of article submission); and (3) publish reports of randomised trials (criteria assessed February 2013). All journals that met the above inclusion criteria were sent an email (February 2013) from the WebCONSORT study scientific committee inviting them to participate in the study. The description of requirements for participation were included in the email and study information sheet (Appendix 1) and editors were asked to verify that they complied with these criteria and that, while they endorsed the CONSORT Statement, they do not actively implement it.

If a journal agreed to participate, and confirmed they met the eligibility criteria, then the journal editor was asked (Appendix 2) to include an electronic web address to the WebCONSORT study website in their request for revision letter to authors for any manuscript identified by the journal as reporting the results of a randomised trial. We did this by asking the journal to include this standard sentence in their revision letter to authors:

“*As part of the process of revising your manuscript we would like you to use the WebCONSORT tool which is designed to help you improve the reporting of your randomised trial. You can access the tool by clicking on the following link: [link to WebCONSORT study site]. Please be aware that by submitting your manuscript to our journal it may be part of a research study, any participation will not impact on any future acceptance or rejection of your manuscript*”.

Participating journals were also informed that we would require access to the revised manuscript to assess reporting quality irrespective of whether it was published or not.

### Random assignment

Authors registering on the WebCONSORT study website were asked to provide some basic information about their randomised trial. This included the name of the journal where the manuscript was submitted, the manuscript number and title, name of submitting author, trial design (e.g. parallel, cluster, non-inferiority, pragmatic), type of intervention (e.g. non-pharmacologic, herbal, acupuncture), and number of study groups (arms). Registered manuscripts were then randomised into two groups (i.e. WebCONSORT tool or control). The sequence of randomisation was computer generated and stratified by whether or not a CONSORT extension was relevant. The assignment was centralised using a web-based system. Authors and journal editors were blinded to allocation of the intervention.

### Interventions

#### Construction of the WebCONSORT tool

**Fig. 1:**
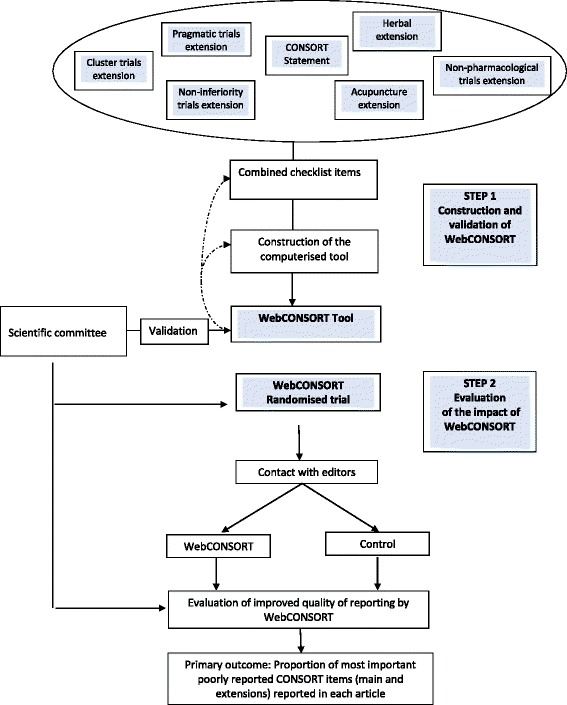
Construction, validation, and evaluation of the WebCONSORT tool

To construct the WebCONSORT tool (Fig. 1) we first combined the different CONSORT extensions to allow grouping of items of similar nature and adaptation of some items to the 2010 version of the CONSORT Statement. Secondly, we designed and built a computerised tool to allow authors to produce a list of items that must be included in the report of their results and a flowchart specific to their trial. The tool combines the main CONSORT checklist and extension checklists for different trial designs (e.g. non-inferiority [18], cluster randomised [19], and pragmatic trials [20]) and for specific types of interventions (e.g. non-pharmacological treatments [21], acupuncture [22], and herbal therapy [23]). The checklist extensions for Abstracts [24] and Harms [25] were not included because they are applicable to all trials. The tool automatically generated a unique list of items customised to a specific trial combining the list of items from the main CONSORT and the items from all relevant extensions (e.g. for a pragmatic trial evaluating a non-pharmacological treatment with cluster randomisation, the main CONSORT checklist was combined with three extensions: pragmatic trial, cluster trial, and non-pharmacological extensions). This list was generated based on the description of the trial made by the author (i.e. type of design and interventions).

A website (Appendix 3: Figure 6) was created where authors were able to log on and register. Using a drop-down menu, they could select their precise type of trial, taking into account the methodological characteristics. Authors were unaware that they were randomised by the software to the WebCONSORT or control intervention.

#### Experimental intervention

Authors randomised to the WebCONSORT arm were directed to a list of CONSORT items specific to their trial which they could print out. They could also obtain an automatic flowchart adapted to the design of their trial. Authors were told that the items generated by the WebCONSORT tool should be reported in the revised manuscript and that the completed checklist and flow diagram should be submitted to the editor. The content of the WebCONSORT tool was validated by members of the study team; this was done by performing a number of “dummy” randomisations to ensure the correctly formatted customised checklist was generated based on different numbers and types of CONSORT extensions being selected. The WebCONSORT tool website was also tested by the scientific committee of the study and by external experts with experience in designing and conducting clinical trials to ensure the website was clear and well understood.

#### Control intervention

Authors randomised to the control group were directed to a dummy version of the WebCONSORT tool website which included the customised flow diagram generator part of the tool but not the main checklist generator or elements relating to CONSORT extensions.

### Outcomes

Our primary outcome was the proportion of the most important and poorly reported CONSORT Statement checklist items (main CONSORT and extensions), pertaining to a given study, reported in the revised manuscript. For the main CONSORT Statement, a group of experts, from within the CONSORT Group, identified the 10 most important and poorly reported CONSORT checklist items to be assessed for each manuscript, based on their expert opinion and supported by empirical evidence where this was available. In addition, the lead authors of each extension were asked to define the five most important and poorly reported modified items specific to their extension (Appendix 4: Table 3). As the number of items differed across trials because the number of relevant extensions varied, we calculated the percentage of possible items that were reported for each article.

The secondary outcomes were the mean proportion of adequately reported items from the main CONSORT Statement (based on the 10 items for the primary outcome above), and the mean proportion of adequately reported items for each of the relevant CONSORT extensions (based on the five items for the primary outcome above). We also collected data on the rejection rate of studies. We had planned to assess the compliance rate of authors submitting a CONSORT checklist to the journal and to obtain feedback from authors and journal editors on the review process; however, these proved difficult to implement in practice and hence were not assessed.

The evaluation of revised manuscripts was conducted by a team of 10 reviewers (based at the Centre for Statistics in Medicine, University of Oxford), with statistical expertise in the design and reporting of clinical trials, working in pairs who were blinded to the nature of the study and allocation of the interventions. Each pair independently extracted data from the manuscripts; any differences between reviewers were resolved by discussion, with the involvement of an arbitrator if necessary. To ensure consistency between reviewers, we first piloted the data extraction form. We discussed any disparities in the interpretation and modified the data extraction form accordingly.

### Sample size

The expected average proportion of adequately reported items in the control arm was 0.60, and our hypothesis was that the proportion of adequately reported items would increase by 25% relatively (15% in absolute value), thus attaining 0.75 in the experimental arm. Assuming a common standard deviation of 0.40, 151 articles per arm were required to demonstrate a significant difference with a power of 90% (two-sided type 1 error is set at 5%), for a total of 302 articles. This sample size calculation was based on the assumption that the mean absolute difference is similar in each stratum (whether or not a CONSORT extension is relevant). We also hypothesized that clustering by journal would have a limited impact because we anticipated the number of journals would be high. Consequently, we did not take into account the clustering by journal in the sample size calculation. We did not anticipate that journals would enroll manuscripts that were not in fact reports of randomised trials.

### Statistical analysis

The main population for analysis were all manuscripts resubmitted to journals after the intervention occurred, which was during the revision process of the manuscript. Statistical analysis was undertaken using STATA IC (version 13). All outcomes were quantitative and described using proportions, mean, standard deviation, and minimum and maximum values. Quantitative variables with asymmetric distributions were presented as medians and interquartile ranges. For the primary and secondary outcomes, we estimated the difference between means in the two groups with 95% confidence intervals. The analysis was also stratified according to those articles which required the inclusion of one or more CONSORT extensions and those which did not. Due to the much larger than anticipated incorrectly specified extensions, we also performed a post-hoc sensitivity analysis for both primary and secondary outcomes to exclude an extension from the analysis of a manuscript if it was wrongly selected by the authors.

## Results

**Fig. 2:**
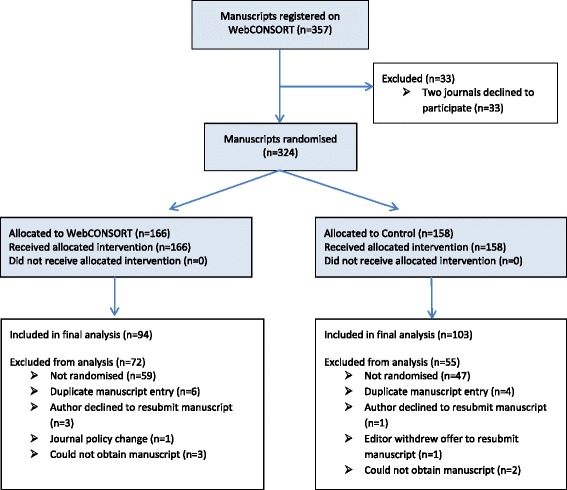
Flow of manuscripts registered on the WebCONSORT study website

Between 25 March 2013 and 22 September 2015, 357 manuscripts were registered on the WebCONSORT study site from 46 general medical and specialty journals with an impact factor ranging from 11.34 to 0.65 as of 2014 (Appendix 5: Table 4). Two journals (*n* = 33 manuscripts) subsequently withdrew and were therefore excluded as we were not able to obtain the revised manuscripts. Of the remaining 324 registered manuscripts, 166 were randomised to the WebCONSORT tool and 158 to the control intervention; of these, 197 were reports of randomised trials (and we were able to obtained the revised manuscript) and were included in the analysis (WebCONSORT *n* = 94; Control *n* = 103). Over a third (39%; *n* = 127) of registered manuscripts were excluded from the analysis. Reasons for exclusion were similar across study arms, the most common reason being that the study was not in fact a report of a randomised trial (Fig. 2). The percentage of eligible manuscripts varied considerably across journals (median 73%; IQR 27% to 100%).

### Characteristics of manuscripts of randomised trials

Of those included in the analysis (*n* = 197), the most common CONSORT extensions selected were non-pharmacologic (WebCONSORT *n* = 43; control *n* = 50), pragmatic (*n* = 20; *n* = 16), cluster (*n* = 10; *n* = 9), and then non-inferiority (*n* = 9; *n* = 8), herbal (*n* = 2; *n* = 13), and acupuncture (*n* = 2; *n* = 0). Over two-thirds (64%; 72%) of manuscripts were registered as requiring one or more CONSORT extension. However, for almost a quarter (23%; 21%) of the manuscripts authors either wrongly selected an extension or failed to select the right extension when registering their manuscript on the WebCONSORT study site (Table 1).

**Table 1:** Number and type of CONSORT extensions (*n* = 197 manuscripts)

	WebCONSORT(*n* = 94)	Control(*n* = 103)
Number of extensions selected^a^		
No extension	34 (36%)	29 (28%)
1 extension	37 (40%)	53 (52%)
2 extensions	21 (22%)	20 (19%)
3 extensions	1 (1%)	1 (1%)
4 extensions	1 (1%)	0
Type of extension selected^b^		
Non-pharmacological extension	43	50
Cluster extension	10	9
Non-inferiority extension	9	8
Pragmatic extension	20	16
Herbal extension	2	13
Acupuncture extension	2	0
Extension correctly matched^c^		
Yes	72 (77%)	82 (80%)
No	22 (23%)	21 (20%)
Reason for mismatch^d^		
Author wrongly selected:		
Pragmatic extension	4	5
Cluster extension	6	4
Non-inferiority extension	3	5
Non-pharmacological extension	2	1
Author failed to select:		
Non-pharmacological extension	10	7
Non-inferiority extension	1	0

^a^Number of extension(s) selected by the author when registering their manuscript on the WebCONSORT randomisation site

^b^Type of extension(s) selected by the author when registering their manuscript on the WebCONSORT randomisation site

^c^Whether extension(s) selected by the author when registering their manuscript was assessed as being the appropriate extension

^d^There may be more than one reason for a miss match between the extension selected by the author and the extension which should have been selected

Most of the 197 trials were two-arm (WebCONSORT 86%; control 82.5%), about half were multicentre (45%; 46.5%), half non-industry funded (50%; 53%), and the median sample size was 98 (IQR, 51 to 180). Around one-third of interventions were drugs (42.5%; 32%), a third were rehabilitation, psychological or educational interventions (30%; 36%), and just under a quarter were surgical or device interventions (23%; 19%). A CONSORT flow diagram was included in 85% and 86% of WebCONSORT and control manuscripts, respectively. Most manuscripts were subsequently published (81%; 84%) in the journal requesting the revision (Table 2).

**Table 2:** General characteristics of manuscripts of randomised trials (*n* = 197 manuscripts)

	WebCONSORT (*n* = 94)	Control (*n* = 103)
Trial design^a^		
Cluster	4 (4%)	4 (4%)
Cross over	2 (2%)	4 (4%)
Factorial	0 (0%)	1 (1%)
Non-inferiority	7 (7%)	3 (3%)
Parallel	88 (94%)	94 (97%)
Pragmatic	15 (16%)	11 (11%)
Split body	1 (1%)	0 (0%)
Disease specialty (top five specialties)		
Nephrology	13 (14%)	15 (15%)
Gastroenterology	12 (13%)	12 (12%)
Obstetrics & Gynaecology	8 (8.5%)	8 (8%)
Psychiatry & Psychology	5 (5%)	8 (8%)
Oncology	7 (7%)	3 (3%)
Type of intervention		
Drug	40 (42.5%)	33 (32%)
Surgery	8 (8.5%)	7 (7%)
Device	14 (15%)	13 (12.5%)
Rehabilitation	5 (5%)	7 (7%)
Psychological	9 (10%)	13 (12.5%)
Education	14 (15%)	17 (16.5%)
Herbal	2 (2%)	13 (12.5%)
Acupuncture	2 (2%)	0
Study centres		
Single	45 (48%)	46 (45.5%)
Multi	42 (45%)	48 (46.5%)
Unclear	7 (7%)	9 (9%)
Number of study groups (arms)		
2	81 (86%)	85 (82.5%)
3	9 (10%)	12 (11.5%)
4	3 (3%)	6 (6%)
> 4	1 (1%)	0
Median sample size (IQR) [parallel group only]	108 (54 to 183)	84 (50 to 157)
Funding		
Solely industry	10 (11%)	11 (11%)
Part industry	9 (9.5%)	6 (6%)
Non industry	47 (50%)	55 (53%)
Unknown	19 (20%)	24 (23%)
None	9 (9.5)	7 (7%)
Flow diagram reported in revised manuscript		
Yes	80 (85%)	89 (86%)
No	14 (15%)	14 (14%)
Manuscript published		
Yes	76 (81%)	87 (84%)
No	18 (19%)	16 (16%)

^a^36/197 (18%) had more than one applicable trial design

### Impact of the WebCONSORT tool on reporting of the revised manuscript

**Fig. 3:**
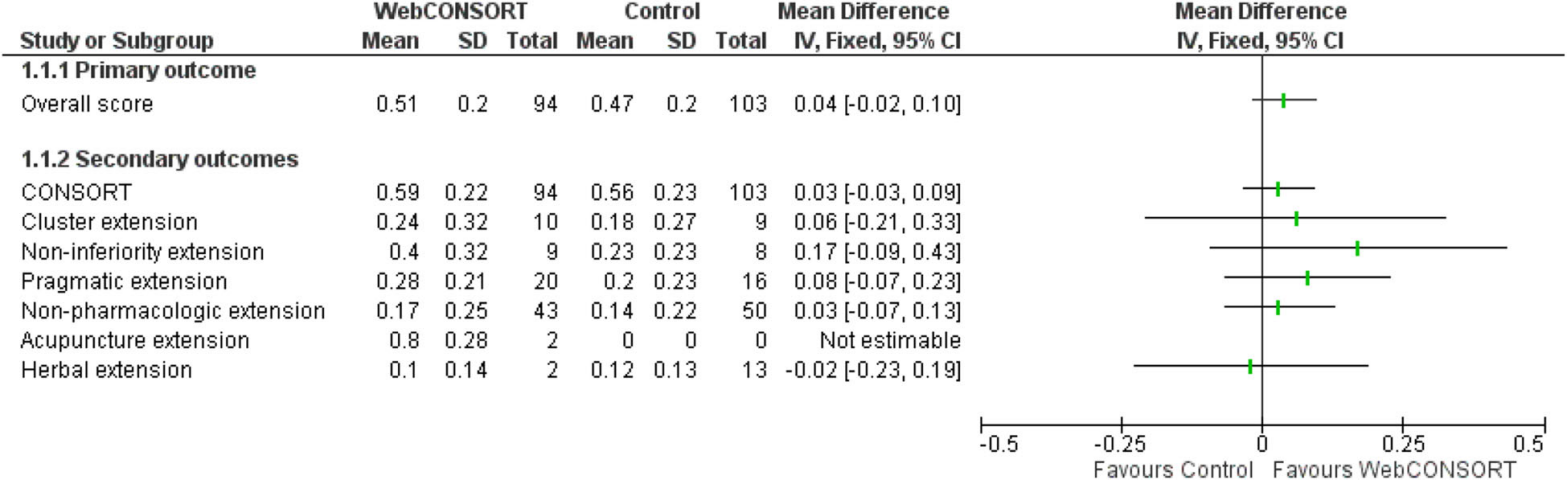
Comparison of overall mean score between WebCONSORT and Control interventions (*n* = 197 manuscripts)

**Fig. 4:**
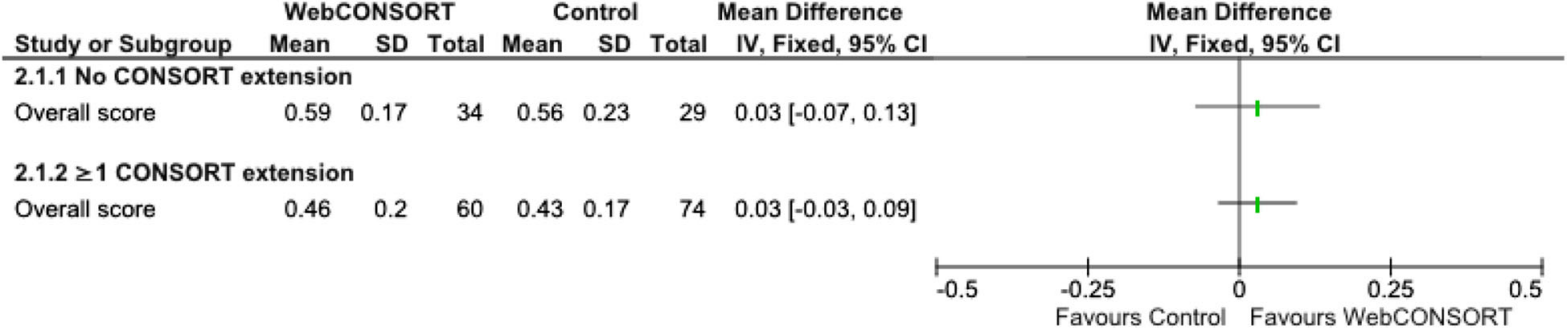
Comparison of overall mean score between WebCONSORT and Control interventions stratified by whether or not one or more CONSORT extensions were selected by the author (*n* = 197 manuscripts)

**Fig. 5:**
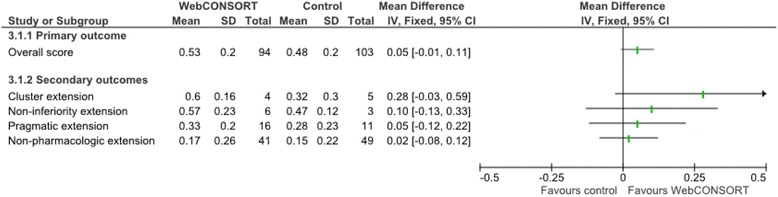
Sensitivity analysis: Comparison of overall mean score between WebCONSORT and Control interventions excluding extensions if wrongly selected by the author (*n* = 197 manuscripts)

**Fig. 6:**
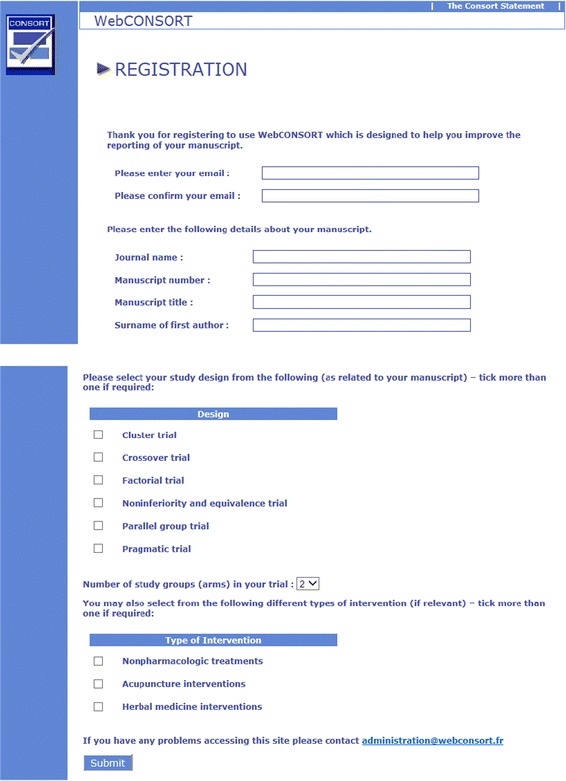
Screen shot of WebCONSORT study website

There was no important difference in the overall mean score (primary outcome) between the WebCONSORT (mean score 0.51; SD 0.2) and control (mean score 0.47; SD 0.2) interventions in the proportion of CONSORT and CONSORT extension items reported pertaining to a given study (mean difference (MD) 0.04; 95% CI −0.02 to 0.10) (Fig. 3). There was no difference between groups when the analysis was stratified according to those articles which were registered as requiring the inclusion of one or more CONSORT extensions (MD 0.03; 95% CI −0.03 to 0.09) and those which did not (MD 0.03; 95% CI −0.07 to 0.13) (Fig. 4), excluding manuscripts for which an extension wrongly selected by the author had little impact on the results (MD 0.05; 95% CI −0.01 to 0.11) (Fig. 5). For the secondary outcomes, there was again minimal difference between groups in the mean proportion of adequately reported CONSORT items (based on the 10 items for the primary outcome) (MD 0.03; 95% CI −0.03 to 0.09) or individual CONSORT extension items (based on the five items for the primary outcome) when analysed separately (Fig. 3). The percentage of adequately reported individual CONSORT and CONSORT extension items (i.e. cluster, non-inferiority, pragmatic, non-pharmacologic, acupuncture, herbal) are given in Appendix 4: Table 3.

## Discussion

### Principal findings and implications

Our study is the first to evaluate the impact of a simple web-based intervention (WebCONSORT) that incorporates the original CONSORT checklist and different CONSORT extensions, on the completeness of reporting of randomised trials published in biomedical journals. Over 40 journals took part in this study, all of which endorsed the CONSORT Statement in their journal ‘Instruction to Authors’. Our study found no overall difference between WebCONSORT and the control intervention in the completeness of reporting of revised manuscripts. This finding suggests that creating a customised CONSORT checklist specific to an individual trial, for use at the revision stage of manuscript submission, may not optimise the use of CONSORT and its extensions.

There are several potential reasons why we did not see an effect of the WebCONSORT intervention. Firstly, all journals included in our sample already endorsed CONSORT, thus authors may have felt they complied with CONSORT guidelines as part of their original submission; although the low level of reporting seen in our study suggest this may not be the case. Secondly, it is possible that the combined customised WebCONSORT checklist had too many items and was overwhelming for authors to comply with. There were also no instructions on how to implement each item in the checklist, suggesting that the checklist alone may not provide sufficient information for most authors. It might be more effective to focus on a core set of CONSORT items with more detail about how to implement each item. Thirdly, it is possible that implementation of a WebCONSORT tool to improve reporting at the revision stage of a manuscript once submitted to a journal may be too late. We may need to intervene earlier in the publication process and provide more explicit succinct guidance along with examples of adequate reporting, tailored to the checklist and context of the trial. COBWEB (Consort-based WEB tool) is an online writing aid tool for authors to use when writing up the results of a randomised trial. The tool consists of a series of text boxes that address CONSORT items, and upon completion the tool provides a formatted Word document. A randomised trial evaluating the impact of COBWEB found that the writing aid tool improved the completeness of manuscripts reporting the results of randomised trials and therefore may be more effective than the creation of a customised checklist [26]; the effectiveness of the writing tool now needs to be tested in a real world setting [27].

The process of conducting our study produced some other interesting findings with important implications. More than one third (39%) of registered manuscripts were excluded from the analysis as they were not reports of randomised trials. This was despite clear instructions provided to journal editorial staff, and included in the revision letter to authors, that only manuscripts reporting the results of randomised trials were eligible for inclusion. Clearly, the journal editorial staff at some journals were unable to correctly identify a randomised trial based on what was reported in the submitted manuscript. Another important finding is that in a quarter (23%) of manuscripts authors either selected an inappropriate CONSORT extension or failed to select the right extension applicable to their trial when registering their manuscript on the WebCONSORT study site. A tool to help authors and journal editors correctly identify the most appropriate checklist to use when reporting the results of a study is currently being piloted by the EQUATOR Network (www.equator-network.org) and may offer a potential solution.

### Comparison with other studies

To our knowledge, our study is the largest randomised trial of its kind, conducted across multiple journals, evaluating the impact of an intervention to improve reporting of published research. Other than the COBWEB study mentioned above [26], very few randomised trials have been conducted evaluating interventions to improve the quality of reporting. One randomised trial evaluated the use of the CONSORT checklist as part of the peer review process and found this could improve the quality of submitted manuscripts [28]; however, this study was only conducted at a single journal*.* Previous studies have tended to explore the impact of the publication of the CONSORT guidelines and CONSORT extensions by studying reporting before and after journal endorsement of CONSORT [17], over time (using a time series analysis) [8, 29] or by monitoring journal endorsement of CONSORT in their ‘Instruction to Authors’ [30].

### Limitations

Our study has several limitations. Firstly, we had to exclude a number of inappropriately registered studies from the analysis and, as such, we had less precision than anticipated to detect a difference between the WebCONSORT intervention and control. Secondly, we do not have information on the number of manuscripts, at each journal, which were eligible for inclusion in the study but where the author chose not to register their manuscript on the WebCONSORT study website (and therefore be randomised). Finally, we do not understand the reasons why authors who registered their manuscript on the WebCONSORT study website and were randomised to the WebCONSORT intervention arm did not then address the recommended checklist items pertaining to their study in their revised manuscript. Future qualitative research to understand the potential barriers and facilitators to better implementation of reporting guidelines would be beneficial.

## Conclusion

Twenty years since its first publication, poor adherence to CONSORT recommendations remains common in published reports of randomised trials. Our randomised trial failed to show a beneficial effect of a customised web-based CONSORT checklist to help authors prepare more complete trial reports. However, it is important to note that the study had less precision than we anticipated to detect a difference due to the exclusion of a large number of inappropriately registered manuscripts. These findings have a number of important implications for future implementation of CONSORT and reporting guidelines more generally. There is a clear need for better education much earlier in the publication process for authors and journal editorial staff on when and how to implement CONSORT and, in particular, CONSORT-related extensions. It may be more effective to focus on a core set of CONSORT items with more detailed information on how to implement each item within the context of a specific trial.

## Appendix 1

### WebCONSORT invitation letter to participating journals

Dear Editor in Chief,

We would like to invite your journal to participate in an exciting new study which aims to help improve the reporting of randomised trials in medical journal articles. We all know that the reporting of clinical trials is not always optimal, despite the impact of reporting guidelines such as the CONSORT Statement, and we recognise that it can be a difficult task for editors to try to improve it.

Our study aims to evaluate whether using a simple web-based tool (WebCONSORT) improves the reporting of randomised trials. The tool allows authors of manuscripts to obtain a customised CONSORT checklist and flow diagram specific to their trial design (e.g. non-inferiority trial, pragmatic trial, cluster trial) and/or type of intervention (pharmacological or non-pharmacological).

We are seeking journals willing to collaborate in this project. The study has been designed so that it will require only a minimal amount of work on your part and yet provide a real opportunity to actively contribute to improving the future reporting of randomised trials, ultimately having a direct impact on patients and patient care. We plan to make the WebCONSORT tool freely available on completion of the study and will of course acknowledge all participating journals in future publications.

We have attached a short summary providing you with more details about the study. If you are interested in participating or have any questions please register your interest by responding to this email. We look forward to hearing from you.

With best wishes

Prof Philippe Ravaud and Dr Sally Hopewell

(Paris Descartes University, France and University of Oxford, UK)

On behalf of the WebCONSORT Steering Committee: Prof Doug Altman (University of Oxford, UK), Dr Ginny Barbour (PLoS Medicine), Prof Isabelle Boutron (Paris Descartes University, France), Dr Agnes Deschartes (Paris Descartes University, France), Dr David Moher (University of Ottawa, Canada), Prof Victor Montori (Mayo Clinc, USA), Dr David Schriger (Annals of Emergency Medicine, USA).

This study is supported by the CONSORT Group and the EQUATOR Network and has been approved by the University of Oxford Central Research Ethics Committee MSD-IDREC-C1-2012-89.

## Appendix 2

### WebCONSORT confirmation letter to participating journals

Dear [insert editor name]

Thank you very much for agreeing to participate in this exciting new study which aims to help improve the reporting of randomised trials in medical journal articles through the use of a simple web-based tool.

To participate in this study we need you include a link to the WebCONSORT study website in your revision letter to authors and you should also notify authors that their manuscript might be part of a research study. The easiest way to do this is to include a standard sentence in your revision letter to authors, for example: “*As part of the process of revising your manuscript we would like to use the WebCONSORT tool which is designed to help you improve the reporting of your randomised trial. You can access the tool by clicking on the following link: [link to WebCONSORT study site]*.” “*Please be aware that by submitting your manuscript to our journal it may be part of research study, any participation will not impact on any future acceptance or rejection of your manuscript.*”

By agreeing to participate in this study, you are thereby agreeing to allow us access to the revised manuscript to assess specific aspects of reporting quality (irrespective of whether the revised manuscript is published). We will contact you periodically through the study with details on any manuscripts registered from your journal. All details of the manuscript content and the identity of its authors will be treated in the strictest confidence.

On completion of the study we plan to make the WebCONSORT tool freely available and will, of course, acknowledge your journal’s contributions in future publications. Thank you once again for your participation in this study, if you have any questions please do not hesitate to contact us.

With best wishes

Prof Philippe Ravaud and Dr Sally Hopewell

(Paris Descartes University, France and University of Oxford, UK)

On behalf of the WebCONSORT Steering Committee: Prof Doug Altman (University of Oxford, UK), Dr Ginny Barbour (PLoS Medicine), Prof Isabelle Boutron (Paris Descartes University, France), Dr Agnes Deschartes (Paris Descartes University, France), Dr David Moher (University of Ottawa, Canada), Prof Victor Montori (Mayo Clinc, USA), and Dr David Schriger (Annals of Emergency Medicine, USA).

This study is supported by the CONSORT Group and the EQUATOR Network and has been approved by the University of Oxford Central Research Ethics Committee MSD-IDREC-C1-2012-89.

## Appendix 4

**Table 3:** Percentage of adequately reported individual CONSORT and CONSORT extension items

CONSORT STATEMENT (10 most important and poorly reported CONSORT items assessed) (*n* = 197 manuscripts)					
Item	Section	CONSORT item	Reported	WebCONSORT (*n* = 94)	Control (*n* = 103)
1	Outcomes (6a)	Completely defined pre-specified primary outcome measure, including how and when they were assessed	Yes	68 (72%)	79 (77%)
2	Sample size (7a)	How sample size determined	Yes	77 (82%)	85 (83%)
3	Sequence generation (8a)	Method used to generate random allocation sequence	Yes	69 (73%)	78 (76%)
4	Allocation concealment (9)	Mechanism used to implement random allocation sequence (such as sequentially numbered containers), describing any steps taken to conceal the sequence until interventions were assigned	Yes	60 (64%)	57 (55%)
5	Blinding (11a)*	If done, who was blinded after assignment to interventions (for example, participants care providers those assessing outcomes)	Yes	44 (47%)	36 (35%)
6	Outcomes and estimation (17a)	For the primary outcome, results for each group, and the estimated effect size and its precision (such as 95% confidence intervals)	Yes	41 (44%)	45 (44%)
7	Harms (19)	All-important harms or unintended effects in each group	Yes	63 (67%)	73 (71%)
8	Registration (23)	Registration number and name of trial registry	Yes	75 (80%)	71 (69%)
9	Protocol (24)	Where trial protocol can be assessed, if available	Yes	21 (22%)	20 (19%)
10	Funding (25)	Sources of funding and other support (such as supply of drugs) and role of funders	Yes	32 (34%)	35 (34%)
(For combined overall score – analysis blinding not applicable refers to where manuscript states not blinded, so scored as yes = 1)					
Flow diagram			Reported	WebCONSORT (*n* = 94)	Control (*n* = 103)
		(flow diagram reported in revised manuscript)	Yes	80 (85%)	89 (86%)
	Participant flow (13a)	For each group, the numbers of participants randomly assigned,	Yes	87 (93%)	99 (96%)
		received intended treatment, and were analysed for the primary outcome	Yes	68 (72%)	82 (80%)
			Yes	81 (86%)	88 (85%)
	(13b)	For each group, losses and exclusions after randomisation, together with reason	Yes	83 (88%)	87 (84%)
CONSORT DESIGN EXTENSIONS (five most important and poorly reported CONSORT items assessed per extension)					
Cluster trials extension selected (*n*=19 manuscripts)					
	Section	Extension item	Reported	WebCONSORT (*n* = 10)	Control (*n* = 9)
1	Background and objectives (2a)	Rationale for using cluster design	Yes	3 (30%)	3 (33%)
2	Sample size (7a)	Method of calculation, number of cluster(s) (and whether equal or unequal cluster sizes are assumed), a coefficient of intra-cluster correlation (ICC or k), and an indication of its uncertainty	Yes	2 (20%)	1 (11%)
3	Randomisation (10b)	Mechanism by which individual participants were included in clusters for the purposes of the trial (such as complete enumeration, random sampling)	Yes	3 (30%)	0 (0%)
4	Statistical methods (12a)	How clustering was taken into account	Yes	4 (40%)	3 (33%)
5	Outcomes and estimation (17a)	Results at individual or cluster level as applicable and a coefficient correlation of ICC or k for each primary outcome	Yes	0 (0%)	1 (11%)
Non-inferiority trials extension selected (*n* = 17 manuscripts)					
	Section	Extension item	Reported	WebCONSORT (*n* = 9)	Control (*n* = 8)
1	Background and objectives (2a & b)	Rationale for using a non-inferiority design Hypothesis concerning non inferiority, specifying the non-inferiority margin with the rationale for its choice	Yes	2 (22%)	1 (12%)
2	Interventions (5)	Whether the reference treatment in the non-inferiority trial is identical (or very similar) to that in any trial(s) that established efficacy	Yes	4 (44%)	0 (0%)
3	Sample size (7a)	Whether the sample size was calculated using non inferiority criterion and, if so, what the non-inferiority margin was	Yes	5 (55%)	3 (37.5%)
4	Statistical methods (12a)	Whether a one- or two-sided confidence interval approach was used	Yes	5 (56%)	4 (50%)
5	Outcomes and estimation (17a)	For the outcome(s) for which non-inferiority was hypothesized, a figure showing confidence intervals and the non-inferiority margin may be useful	Yes	2 (22%)	1 (12%)
Pragmatic trials extension selected (*n* = 36 manuscripts)					
	Section	Extension item	Reported	WebCONSORT (*n* = 20)	Control (*n* = 16)
1	Participants (3)	Eligibility criteria should be explicitly framed to show the degree to which they include typical participants and/or where applicable, typical providers (e.g. nurses), institutions (e.g. hospitals), communities (or localities, e.g. towns) and settings of care (e.g. different healthcare financing systems)	Yes NA	7 (35%) 1 (5%)	2 (12.5%) 3 (19%)
2	Interventions (4)	Describe extra resources added to (or resources removed from) usual settings in order to implement intervention; indicate if efforts were made to standardise the intervention or if the intervention and its delivery were allowed to vary between participants, practitioners, or study sites; describe the comparator in similar detail to the intervention	Yes	7 (35%)	4 (25%)
3	Outcomes (6)	Explain why the chosen outcomes are considered important to those who will use the results of the trial and, when relevant, why the length of follow-up is considered important to those who will use the results of the trial	Yes	2 (10%)	3 (19%)
4	Sample size (7)	If calculated using the smallest difference considered important by the target decision maker audience (the minimally clinically important difference) then report where this difference was obtained	Yes	4 (20%)	3 (19%)
5	Blinding (11)	If blinding was not done, or was not possible, explain why	Yes	4 (20%)	2 (12.5%)
CONSORT INTERVENTION EXTENSIONS (top five items per extension)					
Non-pharmacologic extension selected (*n* = 93 manuscripts)					
	Section	Extension item	Reported	WebCONSORT (*n* = 43)	Control (*n* = 50)
1	Participants (3)	When applicable, eligibility criteria for centres and those performing the interventions	Yes NA	6 (14%) 18 (42%)	4 (8%) 22 (44%)
2	Interventions (4a, b, c)	a) Description of the different components of the interventions and, when applicable, description of the procedure for tailoring the interventions to individual participants b) Details of how the interventions were standardised c) Details of how adherence of care providers with the protocol was assessed or enhanced	Yes	4 (9%)	2 (4%)
3	Sequence generation (8)	When applicable, how care providers were allocated to each trial group	Yes NA	6 (14%) 20 (47%)	3 (6%) 30 (60%)
4	Statistical methods (12)	When applicable, details of whether and how clustering by care providers or centres was addressed	Yes NA	4 (9%) 35 (81%)	5 (10%) 36 (72%)
5	Baseline data (15)	When applicable, a description of care providers (case volume, qualification, expertise, etc.) in each group and centres (volume) in each group	Yes NA	4 (9%) 9 (21%)	5 (10%) 18 (36%)
Acupuncture extension selected (*n* = 2 manuscripts)					
	Section	Extension item	Reported	WebCONSORT (*n* = 2)	Control (*n* = 0)
1	Intervention: Details of needling (2b)	Names (or location if no standard name) of points used (uni/bilateral)	Yes	2 (100%)	0
2	(2c)	Depth of insertion, based on a specified unit of measurement, or on a particular tissue level	Yes	2 (100%)	0
3	(2d)	Response sought (e.g. de qi or muscle twitch response)	Yes	1 (50%)	0
4	(2e)	Needle stimulation (e.g. manual, electrical)	Yes	2 (100%)	0
5	(2f)	Needle retention time	Yes	1 (50%)	0
Herbal extension selected (*n* = 15 manuscripts)					
	Section	Extension item	Reported	WebCONSORT (*n* = 2)	Control (*n* = 13)
1	Intervention: Herbal medicinal product name (4a)	The Latin binomial name together with botanical authority and family name for each herbal ingredient; common name(s) should also be included; the proprietary product name (i.e. brand name) or the extract name (e.g. EGb-761) and the name of the manufacturer of the product; whether the product used is authorised (licensed, registered) in the country in which the study was conducted.	Yes	1 (50%)	5 (38%)
2	Characteristics of the herbal product (4b)	The part(s) of plant used to produce the product or extract. The type of product used (e.g. raw [fresh or dry], extract); the type and concentration of extraction solvent used (e.g. 80% ethanol, 100% H_2_O, 90% glycerin, etc.) and the ratio of herbal drug to extract (e.g. 2 to 1); the method of authentication of raw material (i.e. how done and by whom) and the lot number of the raw material	Yes	0	0
3	Dosage regimen and quantitative description (4c)	The dosage of the product, the duration of administration and how these were determined; the content (e.g. as weight, concentration; may be given as range where appropriate) of all quantified herbal product constituents, both native and added, per dosage unit form; added materials, such as binders, fillers, and other excipients (e.g. 17% maltodextrin, 3% silicon dioxide per capsule), should also be listed	Yes	0	2 (15%)
4	Qualitative testing (4d)	Product’s chemical fingerprint and methods used (equipment and chemical reference standards) and who performed the chemical analysis (e.g. the name of the laboratory used); whether a sample of the product (i.e. retention sample) was retained and if so, where it is Kept or deposited; description of any special testing/purity testing (e.g. heavy metal or other contaminant testing) undertaken, which unwanted components were removed and how (i.e. methods) Standardization: what to standardise (e.g. which chemical components of the product) and how (e.g. chemical processes or biological/functional measures of activity)	Yes	0	0
5	Practitioner (4f)	Description of practitioners (e.g. training and practice experience) part of the intervention	Yes	0	0

## Appendix 5

**Table 4:** Impact factor of journals participating in the WebCONSORT study

Journal	Impact factor (as of 2014)
American Journal of Kidney Diseases	5.90
Annals of Surgery	8.32
Arquivos Brasileiros	1.02
BMC Anesthesiology	1.37
BMC Cancer	3.36
BMC Endocrine Disorders	1.71
BMC Family Practice	1.67
BMC Gastroenterology	2.36
BMC Health Services Research	1.71
BMC Infectious Diseases	2.61
BMC Medicine	7.25
BMC Nursing	–
BMC Oral Health	1.13
BMC Public Health	2.26
BMC Surgery	1.39
British Journal of Geriatrics	–
British Journal of Obstetrics and Gynaecology	3.73
British Journal of Surgery	5.54
Canadian Medical Association Journal	5.96
Child and Adolescent Psychiatry and Mental Health	2.14
Chinese Medicine	1.49
Conflict and Health	–
Critical Care	4.48
Indian Journal of Dermatology	1.34
International Journal of Nursing Studies	2.90
International Journal of Paediatric Dentistry	1.34
Journal of Advanced Nursing	1.74
Journal of Cardiothoracic Surgery	1.04
Journal of Genetic Counseling	2.24
Journal of Gynecologic Oncology	2.49
Journal of Hand Surgery	2.04
Journal of Hepatology	11.34
Journal of the American Podiatric Medical Association	0.65
NIHR HTA monograph	5.12
Neurourology and Urodynamics	2.87
Nordic Journal of Music Therapy	0.96
Orphanet Journal of Rare Diseases	3.36
Pediatric Pulmonology	2.70
Peritoneal Dialysis International	1.53
Physiotherapy	1.91
Public Health Nutrition	2.68
Thrombosis and Haemostasis	4.98
